# Incidental Finding of Appendiceal Mucinous Neoplasm After Trauma: A Case Report

**DOI:** 10.7759/cureus.25832

**Published:** 2022-06-10

**Authors:** Rebecca Odom, Keelin F Roche, Bracken Burns

**Affiliations:** 1 Surgery, Quillen College of Medicine East Tennessee State University, Johnson City, USA

**Keywords:** appendix injury, splenic laceration, peritoneal carcinomatosis index, cytoreductive surgery, appendiceal mucinous neoplasm

## Abstract

Appendiceal neoplasms are uncommon; most are identified by pathologic examination after appendectomy for presumed appendicitis or as an incidental finding. When found incidentally, patients are typically asymptomatic. If the neoplasm has perforated, patients may present with symptoms that mimic acute appendicitis. In advanced disease, patients may have systemic symptoms associated with peritoneal disease, including abdominal distension, weight loss, and diffuse abdominal pain. Because of their rarity, as well as rapidly evolving research on the subject, the nomenclature of appendiceal mucinous neoplasms has proven challenging. This lesion was identified as a low-grade appendiceal mucinous neoplasm (LAMN), previously termed mucoceles or mucinous cystadenomas. LAMNs are non-invasive neoplasms that have the potential to proliferate outside the appendix in a malignant fashion. All mucinous appendiceal neoplasms can perforate and spread mucin production throughout the abdominal cavity, known as pseudomyxoma peritonei (PMP). The presence of PMP designates LAMNs as malignant, though the neoplasm itself is non-invasive. When appendiceal neoplasms have peritoneal involvement, cytoreductive surgery with hyperthermic intraperitoneal chemotherapy (CRS-HIPEC) is the standard of care. Complete cytoreduction has been shown to be an independent predictor of survival. Here we describe a case of a 30-year-old male involved in a motor vehicle collision with a grade IV splenic laceration, who is also found to have a large appendiceal mass. His traumatic injuries required emergent intervention, which delayed treatment of his malignancy.

## Introduction

Incidental findings are common in trauma patients that undergo whole-body computed tomography (CT) scans. Studies on patients receiving whole-body CT scans found incidental findings in 35-50% of patients [1,2]. However, traumatic injuries can complicate the timing and treatment course for an incidentally discovered disease. Here we describe a case of a 30-year-old male involved in a motor vehicle collision who was found to have a large appendiceal mass. He required emergent treatment for a high-grade splenic laceration, which altered and delayed the treatment course of his appendiceal neoplasm. Ultimately, the patient received cytoreductive surgery and hyperthermic intraperitoneal chemotherapy for his low-grade appendiceal mucinous neoplasm and is now disease-free and undergoing monitoring for recurrence. Had this not been found during his traumatic injury it is unknown if he would have received timely treatment for this deadly disease.

## Case presentation

**Figure 1:**
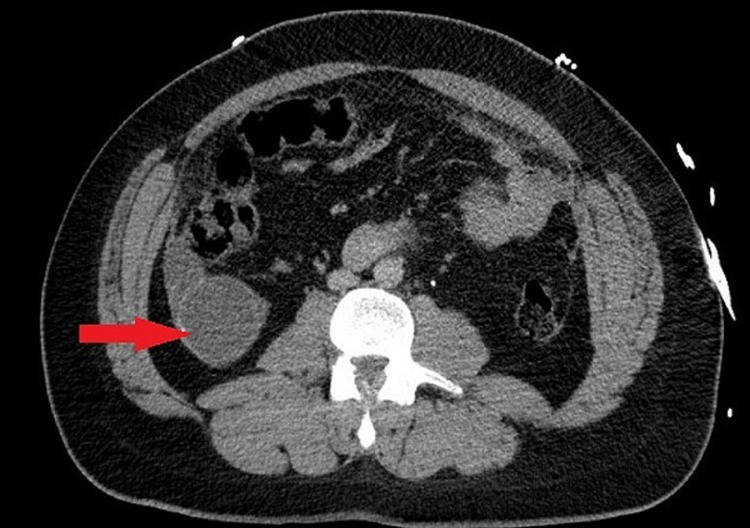
CT demonstrating 5.6 x 4.9 centimeter mass at the tip of the appendix.

A previously healthy 30-year-old male presented to the emergency department after being involved in a motor vehicle collision. Exam and subsequent CT imaging revealed a grade 4 splenic laceration, as well as fractures of the left 6th, 8th, 9th, and 10th ribs, and a chip fracture of the left olecranon. Incidentally, the tip of the appendix was also noted to have a 5.6 x 4.9 cm, peripherally calcified, low-density lesion (Figure 1).

The patient was hemodynamically stable and was taken from the trauma bay to the radiology suite for angioembolization of the proximal splenic artery. Post-procedure, he was admitted to the Intensive Care Unit (ICU) for serial hemoglobin and cardiac monitoring. His hemoglobin trended down slightly from 12.1 to 9.9 g/dL over the course of three days. During this period, a discussion was held with the patient and family by the trauma team regarding his incidental appendiceal lesion - imaging characteristics were consistent with a mucinous neoplasm, though, without a tissue diagnosis, the malignant potential was unknown. He denied having any symptoms prior to admission, any family history of cancer, or ever having a colonoscopy.

**Figure 2:**
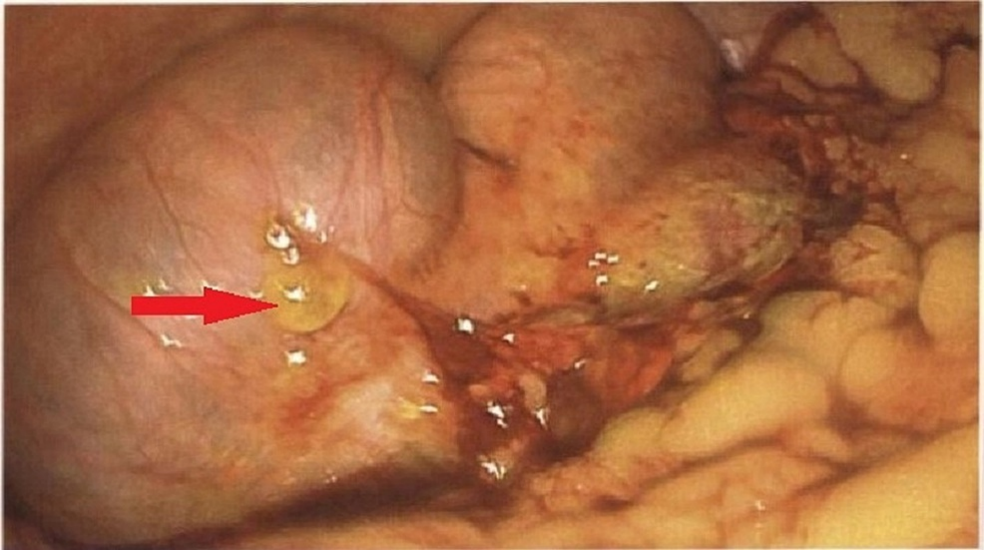
Intraoperative image demonstrating mucinous deposit in the peritoneal cavity.

Seven days after his initial injury when the patient was fully recovered from his splenic embolization and his pain well-controlled from his rib fractures, he was prepared for surgical exploration. At a minimum, the size of the lesion warranted a partial right colectomy. However, this lesion was concerning for a mucinous neoplasm; if mucin were present in the abdomen, he would require hyperthermic intraperitoneal chemotherapy (HIPEC), which would require transfer to a cancer center. The abdomen was entered using the Veress needle technique, and upon inserting the laparoscope into the abdomen, we identified mucin-like material spread throughout the abdomen (Figure 2).

The sequelae of his splenic injury were also noted, with pooling of coagulated blood throughout the abdomen. Additional laparoscopic ports were placed to aid in collecting specimens for pathology. A peritoneal biopsy was obtained for permanent pathology, a sample of mucinous-appearing peritoneal fluid was sent for cytology, and the operation was concluded. Microscopic examination of the peritoneum revealed fibroadipose tissue with reactive mesothelial cells, interstitial mucin, and microcalcifications without evidence of neoplastic cells or overt malignancy. Few atypical epithelioid cells were noted on cytology. Tumor markers obtained prior to surgery demonstrated CA 125 of 23 U/mL (normal 0-35 U/mL), CA 19-9 12 U/mL (normal 0-37 U/mL), CEA 7.2 ng/mL (normal 0-2.5 ng/mL).

Post-operatively, the patient was referred to a facility with HIPEC capabilities due to our finding of pseudomyxoma peritonei. His procedure was postponed for four months to allow for recovery from his polytraumatic injuries and delays related to the COVID-19 pandemic. Prior to surgery, he underwent a colonoscopy in which two tubular adenomas and two hyperplastic polyps were removed; no abnormalities were noted at the appendiceal orifice.

The patient then proceeded with exploratory laparotomy with the intent of cytoreductive surgery, hyperthermic intraperitoneal chemotherapy, and a partial right colectomy. Prior to surgery, cystoscopy and bilateral ureteral stent placement were performed. A midline laparotomy incision was made, and upon entering the abdominal cavity, mucinous deposits were encountered throughout the abdomen - on the surface of the right liver, small bowel mesentery, wall of the anterior rectum, the surface of the terminal ileum, and peritoneum of the right upper quadrant, right lower quadrant, and right mid-abdomen. The peritoneal carcinomatosis index (PCI) score was 13. The appendix was dilated, and the tip had perforated.

A total omentectomy was then performed, followed by resection of the right-sided peritoneum. Mucinous nodules of the liver and anterior rectum were removed bluntly, and nodules of the small bowel mesentery were removed by a combination of sharp dissection and Argon beam. A right partial colectomy with ileocolonic anastomosis was performed, as well as a cholecystectomy. HIPEC was then performed in a standard fashion. No visible disease was noted; the complete cytoreduction (CCR) score was 0. The abdomen was then closed, and surgery was concluded.

The final pathology report revealed a low-grade mucinous neoplasm of the appendix with no invasive component. No further surgical treatment will be necessary at this time. He will undergo continued monitoring through the cancer center.

## Discussion

Appendiceal neoplasms are uncommon; they represent less than 1% of all gastrointestinal malignant neoplasms [3]. They are most often identified by pathologic examination after appendectomy for presumed appendicitis or as an incidental finding. When found incidentally, patients are typically asymptomatic. If the neoplasm has perforated, patients may present with symptoms that mimic acute appendicitis. In advanced disease, patients may have systemic symptoms associated with peritoneal disease, including abdominal distension, weight loss, and diffuse abdominal pain.

Because of their rarity, as well as rapidly evolving research on the subject, the nomenclature of appendiceal mucinous neoplasms has proven challenging. The Peritoneal Surface Oncology Group International (PSOGI) and American Joint Committee on Cancer (AJCC) have only just recently developed guidelines for a more simplified classification of appendiceal neoplasms [4,5]. Epithelial neoplasms are defined as mucinous or nonmucinous; nonmucinous appendiceal adenomas and adenocarcinomas are treated in a pathway identical to similar colon neoplasms [5]. Mucinous neoplasms are further subdivided into non-invasive and invasive neoplasms. Low-grade appendiceal mucinous neoplasms (LAMNs) are non-invasive neoplasms that have the potential to proliferate outside the appendix in a malignant fashion. These lesions were previously termed mucoceles or mucinous cystadenomas, but these terms have fallen out of use.

All mucinous appendiceal neoplasms, both invasive and non-invasive, have the propensity to perforate and spread mucin production throughout the abdominal cavity, known as pseudomyxoma peritonei (PMP) [6,7]. Though LAMNs are indolent, the presence of PMP should be considered malignant, as the disease is now considered disseminated.

When appendiceal neoplasms have peritoneal involvement, cytoreductive surgery with hyperthermic intraperitoneal chemotherapy (CRS-HIPEC) is now considered the standard of care [3,4,8,9]. The Sugarbaker’s Peritoneal Carcinomatosis Index (PCI) is calculated intra-operatively based on the size, location, and number of the tumor deposits and provides an objective measurement of the extent of disease [3]. However, this scoring system has not yet been correlated with survival in cases of complete cytoreduction. Complete cytoreduction, defined as the resection of all visible disease, has been shown to be an independent predictor of survival [3,8,9]. Multiple studies have indicated that patients who undergo CRS-HIPEC for both low-grade and high-grade PMP demonstrate longer patient survival, decrease tumor recurrence, and longer disease-free intervals compared to patients that undergo cytoreductive surgery alone or palliative systemic chemotherapy [8,9]. Systemic chemotherapy has not been shown to be of any benefit to patients with the low-grade disease [2,3].

## Conclusions

Incidental findings are very common in trauma patients and vary tremendously in their clinical significance. This case highlights an incidentally found rare malignancy in a trauma patient. Although his injuries delayed the treatment of the malignancy, the trauma facilitated the diagnosis and without it, it is unknown if he would have been diagnosed in a timely manner. His care was furthered by excellent communication between a rural trauma center and a quaternary care cancer center which allowed him to undergo a lifesaving cancer operation within four months of sustaining his trauma. This case highlights the importance of diligence and a high index of suspicion with incidental findings in trauma which can allow a trauma surgeon to save a patient’s life well beyond the realm of acute injuries.
